# Insight into Coenzyme A cofactor binding and the mechanism of acyl-transfer in an acylating aldehyde dehydrogenase from *Clostridium phytofermentans*

**DOI:** 10.1038/srep22108

**Published:** 2016-02-22

**Authors:** Laura R. Tuck, Kirsten Altenbach, Thiau Fu Ang, Adam D. Crawshaw, Dominic J. Campopiano, David J. Clarke, Jon Marles-Wright

**Affiliations:** 1 Institute of Quantitative Biology, Biochemistry and Biotechnology, The University of Edinburgh, Max Born Crescent, EH9 3BF, UK; 2 Institute for Cell and Molecular Biosciences, Newcastle University, Framlington Place, Newcastle upon Tyne, NE2 4HH, UK; 3 EaStChem, School of Chemistry, The University of Edinburgh, David Brewster Road, Edinburgh, UK, EH9 3FJ, UK

## Abstract

The breakdown of fucose and rhamnose released from plant cell walls by the cellulolytic soil bacterium Clostridium phytofermentans produces toxic aldehyde intermediates. To enable growth on these carbon sources, the pathway for the breakdown of fucose and rhamnose is encapsulated within a bacterial microcompartment (BMC). These proteinaceous organelles sequester the toxic aldehyde intermediates and allow the efficient action of acylating aldehyde dehydrogenase enzymes to produce an acyl-CoA that is ultimately used in substrate-level phosphorylation to produce ATP. Here we analyse the kinetics of the aldehyde dehydrogenase enzyme from the fucose/rhamnose utilisation BMC with different short-chain fatty aldehydes and show that it has activity against substrates with up to six carbon atoms, with optimal activity against propionaldehyde. We have also determined the X-ray crystal structure of this enzyme in complex with CoA and show that the adenine nucleotide of this cofactor is bound in a distinct pocket to the same group in NAD^+^. This work is the first report of the structure of CoA bound to an aldehyde dehydrogenase enzyme and our crystallographic model provides important insight into the differences within the active site that distinguish the acylating from non-acylating aldehyde dehydrogenase enzymes.

Bacterial microcompartments (BMCs) are protein-walled metabolic compartments that sequester pathways for the catabolism of various carbon sources^1^,^2^. The protein shells of BMCs are formed through the interaction of thousands of copies of different proteins belonging to the BMC protein family^3^ to produce a pseudo-icosahedral container of around 150 nm in diameter. The enzyme contents of BMCs are directed to the interior of the compartment as it forms, through interactions between short peptides appended to their functional domains and the BMC shell proteins^4^,^5^.

BMCs are found in species with diverse ecological niches, from pathogenic strains of *Escherichia coli* and *Salmonella*^6^,^7^, to the cellulose degrading *Clostridium phytofermentans*^8^, and marine bacteria including *Haliangium ochraceum* and *Rhodospirillum rubrum*^9^. As a consequence of this wide species distribution, BMCs have diverged in substrate specificity and the enzyme activities that they encapsulate to be able to utilise carbon sources such as 1,2-propanediol^7^,^10^, ethanolamine^11^,^12^ fucose/rhamnose^13^, and choline^14^. A common feature of the metabolic pathways found within BMCs is the production of a toxic aldehyde intermediate in the breakdown of their substrates^6^,^10^,^15^. In the well-characterised propanediol utilisation BMC, a vitamin B12 dependent lyase enzyme acts on the 1,2-propanediol substrate to produce propionaldehyde^10^; while the lyase in the ethanolamine utilisation BMC produces acetaldehyde and ammonia as its products^16^. A recently discovered family of BMCs use a glycyl-radical enzyme (GRE) to break down their primary substrates producing an aldehyde intermediate, such as the production of acetaldehyde from the choline utilisation BMC found in *Desulfovibrio desulfuricans*^14^ and the *C. phytofermentans* fucose/rhamnose BMC^13^. To detoxify the aldehyde produced by these enzymes, and to ultimately generate ATP through substrate level phosphorylation, all BMCs identified to date possess an acylating-aldehyde dehydrogenase enzyme that produces an acyl-CoenzymeA and NADH^15^.

The aldehyde dehydrogenase enzymes (AldDH) encoded with BMC loci are invariably accompanied by an alcohol dehydrogenase enzyme^17^, the activity of which appears to be necessary to recycle the NADH produced by the activity of the aldehyde dehydrogenase. Genetic knockout of the aldehyde dehydrogenase in the propanediol^17^ and ethanolamine^18^ BMC loci leads to reduced growth on their respective substrates. These results imply that BMCs contain private NAD^+^/NADH cofactor pools that are not exchanged with the bulk cytosol of the host cell and explain the requirement for both the aldehyde and alcohol dehydrogenase enzymes to be present within the BMC. As a consequence of the need to maintain the balance of the cofactor pool within the BMC, two substrate molecules are required to produce one acyl-CoA molecule, with the second substrate molecule used to oxidise the NADH produced by the AldDH through the action of the alcohol dehydrogenase to produce alcohol from the aldehyde (Supplementary Fig. 1). This is consistent with the production of almost equimolar amounts of the carboxylic acid product from substrate-level phosphorylation via an acyl-phosphate intermediate, and the alcohol product of the alcohol dehydrogenase^13^.

The aldehyde dehydrogenase superfamily are well studied and are active against a range of aldehydes, including the short chain fatty aldehyde products of the lyase enzymes in BMCs^19^,^20^,^21^. These enzymes have a common architecture, with a Rossman-fold nucleotide-binding domain that positions the NAD(P)^+^ cofactor required for hydride transfer from the aldehyde substrate^22^; the catalytic domain has a substrate-binding tunnel with a catalytic cysteine residue and glutamic acid residue that acts as a general base in the hydrolysis of the acyl-enzyme intermediate^23^. The acylating aldehyde dehydrogenase enzymes do not possess the glutamic acid general base residue, presumably because the CoA cofactor acts to resolve the acyl-enzyme intermediate to produce the acyl-CoA product via a bi-uni-uni-uni-ping-pong mechanism^24^. Although a recent study of the kinetics of the PduP enzyme from *Lactobacillus reuterii* with its native substrate presented a model of CoA binding to the enzyme in the same binding pocket as NAD^+^, there are no experimental structures of acylating-aldehyde dehydrogenase enzymes in complex with CoA in the PDB^25^.

Here, we study the kinetics and substrate specificity of an AldDH enzyme from the *Clostridium phytofermentans* fucose/rhamnose utilisation BMC, Cphy1178. We present the X-ray crystal structure of an N-terminal truncation of this enzyme and show by native mass spectrometry that the quaternary structure of the protein is a tetramer. We have determined the structure of the protein in complex with its cofactors NAD^+^ and CoA and show that the adenine nucleotides of these co-factors adopt different conformations within the Rossman fold domain.[Table t1]

## Results

### Aldehyde dehydrogenase activity of Cphy1178

Using recombinantly produced protein, we tested the NAD(P)^+^ dependent *in vitro* activity of the putative aldehyde dehydrogenase enzymes from the three *Clostridium phytofermentans* BMC loci (Cphy1178, Cphy1428, Cphy2642) and the *Clostridium difficile* ethanolamine utilisation locus (CD630_19170). The full-length recombinant enzymes produced for this study were unstable in various common buffer systems and aggregated rapidly upon purification; therefore, we produced truncations to remove the proposed BMC localisation sequences (Supplementary Fig. 2). These truncated enzymes were more stable in solution than their full-length counterparts and were used in subsequent activity assays.

By monitoring the hydride transfer step of the aldehyde dehydrogenase enzyme reaction by spectrophotometric measurement of the production of NADH in the presence of aldehyde substrates, we were only able to detect NAD^+^ dependent activity for Cphy1178. The other enzymes displayed no distinguishable activity with either NAD^+^, or NADP^+^, for the substrates used in this study. This may be due to issues with the instability of these proteins outwith their native environment within the BMC, or a requirement for some additional factor not present in our assays. The Cphy1178 aldehyde dehydrogenase displayed negligible activity with glyceraldehyde as a substrate. However, we were able to detect activity against short-chain fatty aldehydes with up to seven carbon atoms, although longer chain aldehydes were difficult to assay reliably due to their insolubility in biological buffers and we were unable to determine accurate kinetic parameters for C7 and higher aldehydes (Table 2 and Supplementary Fig. 3). Cphy1178 enzyme displayed the lowest *K*_M_ and the highest *k*_cat_/*K*_M_ values for the substrates tested. This data is consistent with the hypothesis that this protein acts as a propionaldehyde dehydrogenase within the *C. phytofermentans* fucose/rhamnose BMC. It is noteworthy that this enzyme displays substrate inhibition at high concentrations of aldehydes; interestingly this is more marked with aldehydes with an odd number of carbon atoms. This substrate inhibition is consistent with previous reports on the activity of yeast ALDH2 enzyme^19^.

Our observation that this enzyme displays good activity against longer chain aldehydes offers promise for engineering BMCs to produce alkanes and make use of the native aldehyde dehydrogenase enzymes found within them^26^. Based on identification of the catalytic cysteine by sequence alignment, mutagenesis of C269A completely abolished enzymatic activity, as did the mutagenesis of the putative base (H387)^27^. The structures of these two mutants were essentially unchanged compared to the wild-type enzyme, indicating that changes to their catalytic activity were not a consequence of protein misfolding (see below for detail of the structure of this protein).

**Figure 1:**
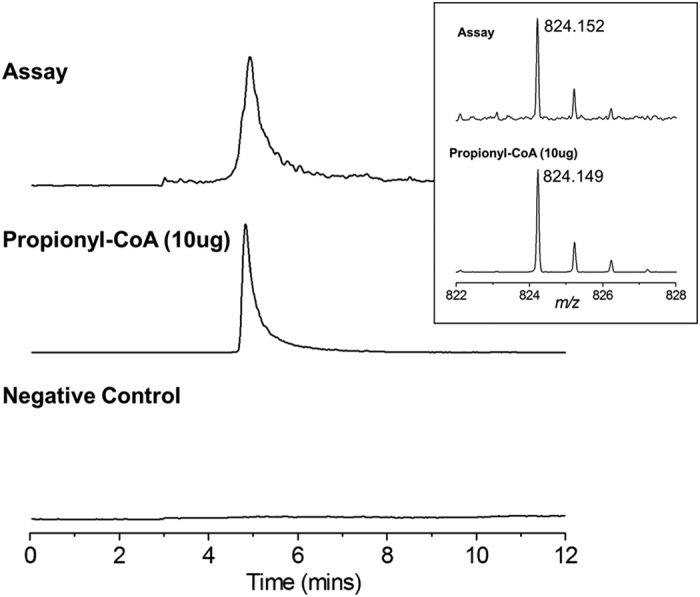
Detection of propionyl-CoA assay product by LC-MS. *Main Figure*, Extracted ion chromatogram at 824 *m/z*. *Top*, the Cphy1178_(20–462)_ assay mixture containing (50 nM Cphy1178_(20–462)_, 75 μM NAD^+^, 100 μM CoA, 10 mM propionaldehyde, 100 mM Tris.HCl pH 8.0, 100 mM KCl); *middle*, 10 ug propionyl-CoA control; *bottom*, negative control. *Insert*, mass spectrum of the Propionyl-CoA obtained by summing the spectra between 4.8 to 5.8 minutes. *Top*, Cphy1178_(20–462)_ assay; *bottom*, propionyl-CoA control. (predicted monoisotopic mass [M+H]^+^ C_24_H_41_N_7_O_17_P_3_S; 824.1487 Da).

It is important to note, that our spectrophotometric assay only monitors the production of NADH which occurs in the first step of the multistep reaction. In order to confirm the acyl-transfer from the acyl-enzyme intermediate to CoA we used mass spectrometry to detect the final product. This MS assay was performed using propionaldehyde as the substrate, with reaction mixtures containing NAD^+^ and CoA. LC-MS analysis confirmed the presence of the propionyl-CoA product (Fig. 1) at a mass consistent with the monoisotopic mass of the standard used as a control.

### Structure of Cphy1178_(20–462)_

**Figure 2:**
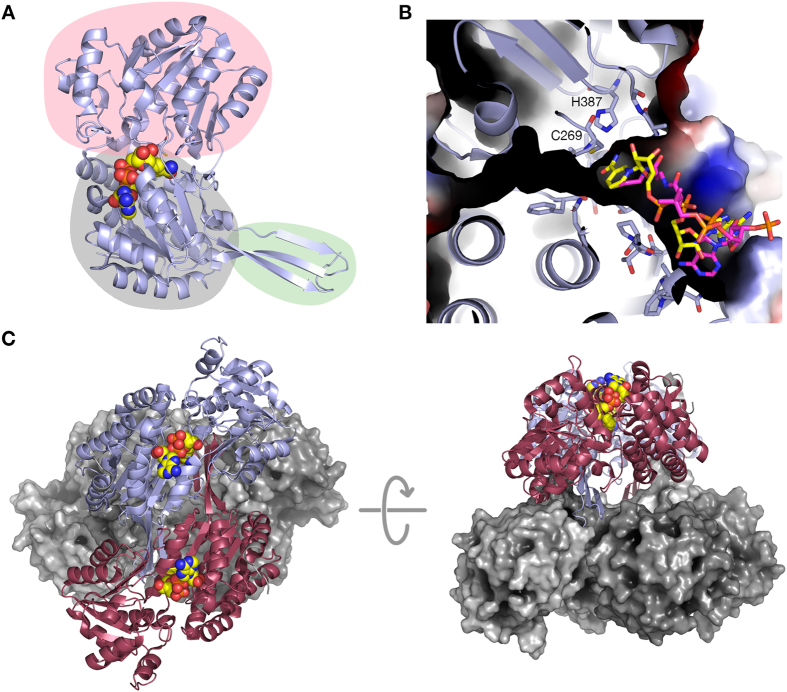
Structure of Cphy1178_20–462_. (**A**) Secondary structure cartoon of a monomer of Cphy1178_20–462_ showing bound NAD^+^ as spheres coloured yellow for carbon, red for oxygen, blue for nitrogen and orange for phosphorus. The catalytic domain is highlighted in pink, rossman fold nucleotide binding domain in grey and oligomerisation domain in green. (**B**) Active site tunnel and nucleotide binding pocket. The surface of Cphy1178_20–462_ is shown coloured by electrostatic potential (blue for positive, red for negative) and the secondary structure cartoon is shown in blue. The catalytic cysteine and histidine residues are shown as sticks. Bound cofactors, NAD^+^ and CoA are shown with yellow and pink carbons respectively. (**C**) Cphy1178 forms a dimer of dimers quaternary structure. Two subunits are shown as cartoons, with NAD^+^ shown as spheres to show the relative orientation of the nucleotide-binding cleft; two further subunits are shown as grey surface representations.

To understand the co-factor binding properties of Cphy1178 we determined the structure of an N-terminal truncation, comprising residues 20–462, with bound NAD^+^ cofactor to 1.6 Å resolution. In these structures, the asymmetric unit contains a single chain with residues 28 to 462 visible in the electron density map. The overall structure corresponds to other members in the aldehyde dehydrogenase family (Fig. 2A), with a catalytic domain (residues 238–427, highlighted pink), a cofactor-binding domain with the Rossman-fold type nucleotide binding architecture (residues 28–108, 127–237, and 428–447, highlighted grey), and an oligomerisation domain (residues 109–126 and 448–462, highlighted green). The catalytic and nucleotide binding domains come together to form an extended nucleotide and ligand-binding tunnel that is open at both ends, with the catalytic cysteine (C269) at the centre of the tunnel and the NAD^+^ cofactor binding at one side (Fig. 2B). The ligand-binding tunnel is about 5 Å in diameter at its widest point and spans 16 Å from the solvent exposed entry point to the catalytic cysteine and is lined with hydrophobic residues. The tunnel is long enough to accommodate up to a C10 aldehyde, although *in vivo* the enzyme is unlikely to encounter such a substrate. The structures of Cphy1178 were also solved with mutations in active site residues, C269A with bound CoA (1.77 Å resolution) and H387A (2.08 Å resolution) to ensure that mutation of these residues did not destabilise the structure of the protein. Both of these proteins display essentially identical structures to the wild-type protein. In the structure of the Cphy1178_C269A_ mutant the position of the loop between 335 and 339 was not clear in the electron density maps, so was omitted from the final structure refinement. This structure was determined with CoA bound in the active site and is discussed below in the section on cofactor binding.

**Figure 3:**
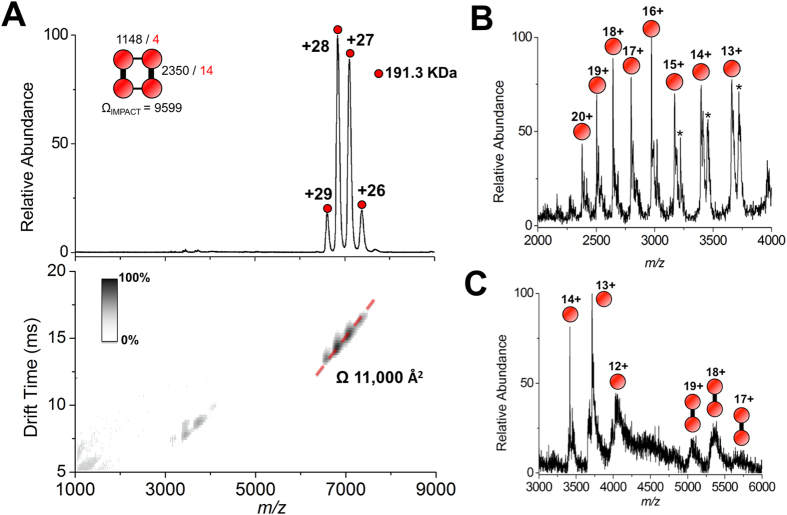
Native mass spectrometry analysis of Cphy1178. (**A**) Ion-mobility MS analysis of recombinant Cphy1178 displays a charge state distribution consistent with the +26 to +29 ions of a tetrameric assembly of Cphy1178 (*Top*, 191.3 KDa). Ion-mobility data, displayed as a greyscale heat map (*bottom*) reveals that each charge states exhibit a single drift time. When corrected for charge, the drift times of all charge states are all consistent with a collision cross section of 11,000 Å^2^ (hashed red line). (**A***, Insert*) A topological cartoon of the Cphy1178 tetramer using ball-and-stick representations (ball, monomer; stick, interface). Interface areas in Å^2^ (black) and number of salt bridges (red) are marked; and calculated collision cross section (Ω) in Å^2^ is given. (**B**) Gas phase dissociation of the Cphy1178 using collision induced dissociation leads exclusively to the appearance of monomeric Cphy1178; a 1 KDa adduct is also observed (*) (**C**) Partial disruption of the Cphy1178 assembly by solution-phase dissociation prior to MS analysis (using 40% v/v MeOH) leads to the appearance of both monomeric and dimeric subcomplexes.

Cphy1178 forms a tetrameric quaternary structure generated by crystal symmetry, with molecules related by D2 symmetry (Fig. 2C). A tetrameric assembly is also observed when analysing Cphy1178 by native (nondenaturing) electrospray ion-mobility mass spectrometry (IM-MS)^28^; using this technique a single charge state distribution is observed which corresponds to tetrameric Cphy1178 in the +26 to +29 charge states (191.3 kDa assembly mass, in agreement with the predicted molecular mass of 4 × 47,452 Da; Fig. 3A). Ion-mobility of the Cphy1178_(20–426)_ tetrameric assembly reveals that the complex exists as a single conformer with a collision cross section (CCS) of ~11,000 Å^2^ (Fig. 3A). This gas-phase value is in agreement with the calculated CCS of the tetrameric structure determined by crystallography (Cphy1178_28–426_; CCS calculated as 9599 Å^2^ by IMPACT v 0.9.1); suggesting that the overall solution architecture of the enzyme is retained during MS analysis. This tetramer also corresponds to the most probable stable arrangement proposed by the PISA server^29^ and is consistent with previously published structures of this family of enzymes^23^.

From the crystal structure it is apparent that intersubunit interactions are primarily mediated by contacts between the oligomerisation domain and the catalytic domain, which produces a dimer with 2350 Å^2^ buried surface per monomer (out of a total solvent accessible surface of 18,229 Å^2^) and is stabilised by 15 hydrogen bonds and 14 salt bridges (interface between red/blue and grey/grey monomers in Fig. 2C). A second dimerisation interface is found between symmetry related oligomerisation domains, this is stabilised by 3 hydrogen bonds and 4 salt bridges and buries 1148 Å^2^ of surface area per monomer (red/grey and blue/grey monomers in Fig. 2C). These dimers are further stabilised by contacts between an α-helix in the catalytic domain (residues 395–407) and the oligomerisation domain (500 Å^2^ buried SA, one hydrogen bond and two salt bridges); the C-terminus of the protein is buried within this interface and the terminal carboxylic acid group is visible in the electron density map.

Further evidence for the existence of two interfaces of differing strengths within the Cphy1178 tetramer came from topology-mapping mass spectrometry studies^30^,^31^. Collision induced dissociation (CID) of the protein tetramer led to the appearance of a charge state distribution consistent with monomeric Cphy1178 (Fig. 3B) – an observation consistent with the *typical* mechanism of dissociation using this technique (i.e. ejection of a highly charged monomer from the assembly)^32^. In contrast, titration of increasing organic solvent to the protein solution prior to MS analysis, resulted in the appearance of both monomer and dimer charge state distribution in the mass spectrum (Fig. 3C). Solution-phase dissociation of protein complexes is known to occur via dissociation of the weakest interface in the complex first. Therefore using this technique, subcomplexes of the protein assembly can be formed, their composition determined by MS, and relative interface binding strengths can be inferred^33^. We interpret our observation as cleavage of the second smaller interface, whilst the larger interface (area 2350 Å^2^; which includes extensive salt-bridge interactions) remains intact.

### Co-factor binding

**Figure 4:**
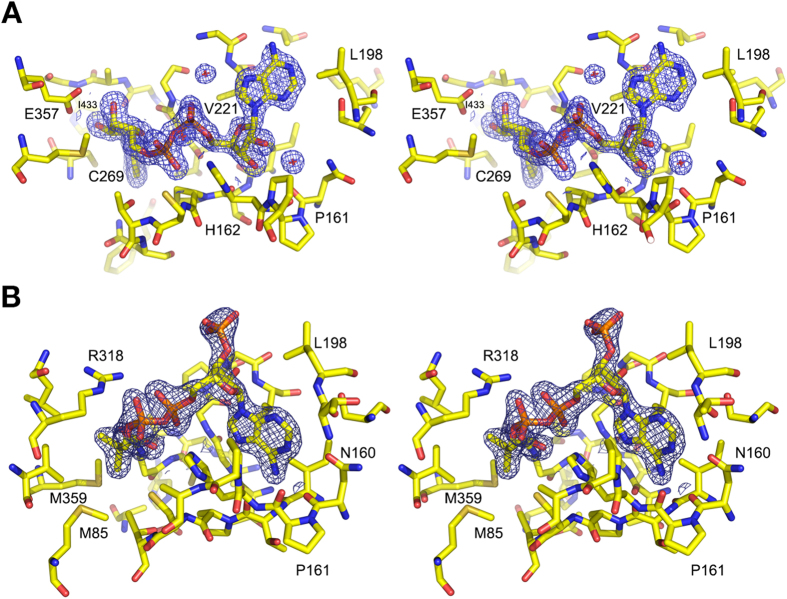
Electron density maps of bound NAD^+^ and CoA cofactors. (**A**) Structure of NAD^+^ soaked Cphy1178_20–462_. NAD^+^ and protein residues within 4 Å shown as stick representations and 2mFo-DFc map shown as a blue mesh contoured at 1σ. (**B**) Structure of CoA soaked Cphy1178_20–462(C269A)_ displayed as in **(A)**.

The structure of Cphy1178_20–462_ was determined with NAD^+^ bound in the nucleotide binding cleft and active-site pocket after soaking apo-crystals with crystallization solution supplemented with 10 mM NAD^+^. The ligand displayed excellent electron density (Fig. 4A) and was refined to an occupancy of 0.83 in the final structure using phenix.refine. The NAD^+^ is found in the hydride transfer conformation^34^,^35^ and participates in 4 direct hydrogen bonding interactions with the enzyme and a number of other interactions mediated by ordered solvent molecules. The adenine ring does not directly participate in any hydrogen bonding interactions, but is positioned between Leucine 198 and Valine 221. The N7 of the adenine ring accepts a hydrogen bond from an ordered solvent molecule that is also coordinated by an oxygen atom in the adenosine phosphate group and the backbone nitrogen of Valine 221. Both O2 and O3 of the adenosine ribose interact with an ordered water molecule that bridges them to the carbonyl oxygen of Proline 161. Similarly, two oxygen atoms from the phosphate groups are bridged to Histidine 162 via a solvent molecule. Glutamine 357 participates in hydrogen bonding interactions with O2 and O3 of the nicotinamide ribose. The nicotinamide ring is positioned next to the catalytic Cysteine 269 through hydrogen bonding interactions between N7 and O7 and the peptide backbone of Isoleucine 433. The absence of enzymatic activity in the presence of the NADP^+^ co-factor is explained by the presence of a histidine and proline residue blocking the position that the 2′-phosphate adopts in structures of aldehyde dehydrogenase enzymes determined with NADP^+^ bound (Supplementary Fig. 4A/B).

The structure of Cphy1178_20–462(C269A)_ was determined using crystals soaked with 10 mM CoA. The cofactor showed good electron density for the adenosine group, but poor density for the pantothenic acid group, particularly the terminal region with the sulphydryl group in the active site, which has no visible electron density (Fig. 4B). A comparison of the structure of the NAD^+^ and CoA bound forms of the protein show that the structures are essentially identical, with an rmsd Cα of 0.24 Å over 431 residues. The two structures differ only in the position of the loop between residues 215 and 223, where P219, G220 and V221 are shifted toward the adenine ring in the NAD^+^-bound structure (Supplementary Fig. 5).

In contrast to the models of CoA binding to the *Pseudomonas* DmpF aldehyde dehydrogenase^36^ and *Lactobacillus reuteri* PduP aldehyde dehydrogenase^25^ our crystal structure shows distinct adenine-binding modes for NAD^+^ and CoA (Fig. 4B and Supplementary Fig. 4C–F). The adenine ring is flipped by 180 degrees relative to its position in the NAD^+^ structure and makes hydrogen-bonding contacts with both the side chain and carbonyl oxygen of Asparagine 160, and the carbonyl oxygen of Threonine 134, placing the phospho-ribose group above both of the nucleotide binding pockets. The phosphate groups between the nucleotide and pantothenate group are held in place by hydrogen bonds formed between the side-chain nitrogen atoms of Histidine 162 and Arginine 318. While there is clear electron density for the nucleotide and phosphate-proximal portion of the pantothenate group of the CoA, the β-mercapto ethylamine group is not visible, indicating that the distal arm of CoA is flexible in the absence of thio-acyl substrates bound to the catalytic cysteine for acyl transfer.

## Discussion

### Substrate Discrimination

**Figure 5:**
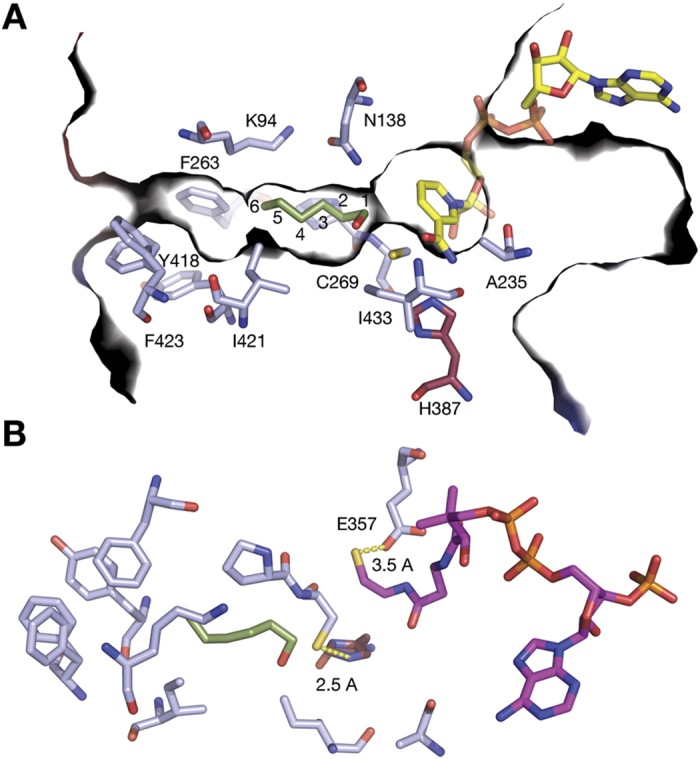
Cphy1178_20–462_ active site architecture. (**A**) Surface slice through Cphy1178_20–462_ showing the position of NAD^+^ and modelled hexanal substrate. Protein residues and substrates shown as stick representations with different coloured carbon atoms, the active-site histidine is shown in red for clarity. The ligand/substrate-binding tunnel is open at both ends and accommodates the NAD^+^/CoA cofactors at one end and the aldehyde ligand at the other. The aldehyde-binding portion of the tunnel is lined with hydrophobic residues and is gated by F423, which is present in multiple conformations in the crystal structure. (**B**) Binding of CoA in the active site showing distances between H387 and C269 and the sulphur of the CoA and E357. CoA shown in magenta.

Our initial enzyme assays indicate that Cphy1178_20–426_ is active against a range of short chain aldehydes (C2–6). This observation can be rationalised by analysis of the structure of the enzyme, which reveals that the substrate-binding cleft of Cphy1178_20–426_ is lined with hydrophobic residues and can comfortably accommodate an aldehyde substrate with up to six carbon atoms and could potentially fit a chain of up to nine or ten carbon atoms in the substrate tunnel (Fig. 5A). A phenyl alanine residue (F423) found at the solvent exposed side of the tunnel appears in multiple conformations in the crystal structure, this may indicate that it acts to control substrate access at this entry site. The tunnel is enlarged close to the catalytic cysteine, which may allow substrate rearrangement during acyl-transfer. The lack of activity against glyceraldehyde is explained by the presence of hydrophobic residues in this region (I421 and I433), which would make unfavourable interactions with the hydroxyl groups of this substrate. Cphy1178 is present in the *C. phytofermentans* fucose/rhamnose BMC, which has a fucose aldolase enzyme that produces lactaldehyde (2-hydroxypropanal) as its product, alongside a propanediol dehydratase that produces propionaldehyde^13^. Comparison of the ligand-binding tunnel of Cphy1178 with the lactaldehyde dehydrogenase enzyme from *Escherichia coli* (PDBID: 2HG2), shows that the two isoleucine residues in the substrate-binding tunnel of Cphy1178 are replaced by a glutamic acid and a histidine residue to accommodate the polar hydroxyl group of its lactaldehyde substrate^23^. These key amino acid differences and the preferential activity of Cphy1178 against short-chain fatty aldehydes indicate that propionaldehyde is the most likely natural substrate for Cphy1178.

### Acylation

**Figure 6:**
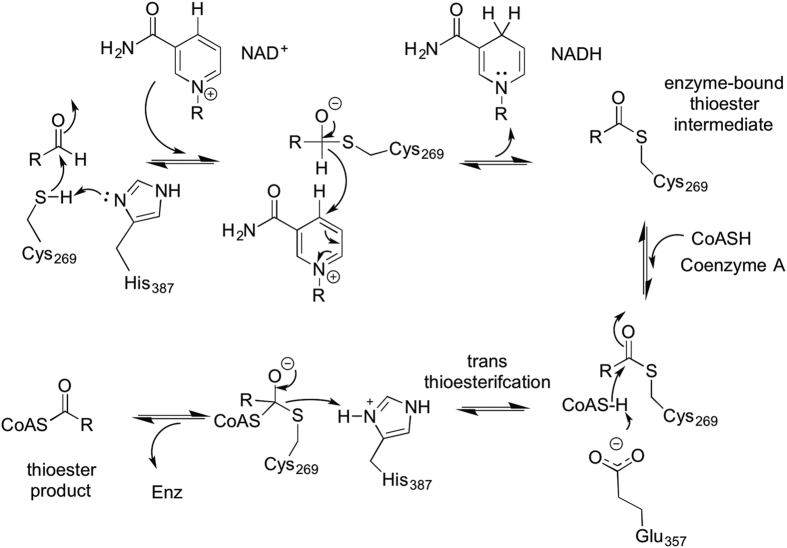
Proposed catalytic mechanism of Cphy1178. The catalytic cycle proceeds via a bi-uni-uni-uni-ping-pong mechanism involving acylation of the catalytic cysteine (C269) and hydride transfer to NAD^+^, followed by trans-thioesterification between the enzyme and CoA to produce the Acyl-CoA product.

We have shown that h1178 shows a higher level of activity with propionaldehyde as a substrate compared to other aldehydes tested and is capable of acyl-transfer to CoA using this substrate. We have also determined the structure of this enzyme with CoA bound in the active site. The acylating aldehyde dehydrogenase family enzymes do not possess the glutamic acid general-base that is required to activate the catalytic cysteine and to deprotonate water for deacylation of the acyl-enzyme intermediate^37^. In Cphy1178 this residue is replaced by an alanine (A235) (Fig. 5A) and is a small hydrophobic residue in all of the acylating aldehyde dehydrogenases in this study (Supplementary Fig. 2). Mutagenesis of a strictly conserved Histidine residue (H387), that is within 2.5 Å of the catalytic cysteine (Fig. 5B) completely abolishes activity of the enzyme. This residue is not found in any of the non-acylating aldehyde dehydrogenase enzymes^20^. The proximity of this residue to the catalytic cysteine is incompatible with the formation of the correct geometry for deprotonation of water for resolution of the acyl-intermediate to produce a carboxylic acid product^34^. In light of our biochemical data showing that the H387A mutant is inactive, we suggest that this residue acts as a base to activate the catalytic cysteine and to stabilise the acyl-transfer intermediate between the enzyme and CoA cofactor. Due to the proximity of a glutamic acid residue (E357) to the cysteine group of CoA (Fig. 5B) and the strict conservation of this residue, we propose a model in which this residue activates the CoA cofactor for acyl transfer from the acyl-enzyme intermediate (Fig. 6). In our reaction scheme H387 deprotonates the catalytic cysteine to allow nucleophilic attack on the substrate to form tetrahedral intermediate. Consistent with previous reports the oxyanion would be N138^34^. After hydride transfer to NAD^+^, the NADH product leaves the cofactor-binding pocket in the rate-limiting step, followed by entry of CoA, which is deprotonated by E357 and subsequently attacks the acyl-enzyme intermediate to produce the thioester product and free enzyme (Fig. 6). The extra proton on E357 is likely transferred to bulk water via a proton-relay system comprising the conserved residues N138 and K94^38^.

In this study we have determined the activity of Cphy1178 against a range of aldehyde substrates and present the first structure of CoA bound to an acylating aldehyde dehydrogenase and suggest a role for a conserved histidine found in place of the glutamic acid that acts as the general base in non-acylating aldehyde dehydrogenases. The capture of acyl-transfer intermediates in future crystal structural studies will shed further light on the mechanism of acyl transfer in this important class of enzymes that are essential to the function of bacterial microcompartments.

## Materials and Methods

### Cloning

Genes encoding aldehyde dehydrogenase enzymes from the *Clostridium phytofermentans* and *Clostridium difficile* bacterial microcompartments were amplified by PCR using the primers detailed in Table 2 using the KOD HotStart DNA polymerase as described in the user protocol. PCR products were run on a 0.8% Agarose/TAE gel and visualised by SybrSafe staining (Life Technologies), bands of the appropriate size for each construct were excised and subjected to gel-cleanup using a Qiagen kit[Table t2]and following the protocol described in the user manual. Purified PCR products were digested with appropriate restriction enzymes (Fermentas) for ligation into the pET28a vector (Novagen). Site directed mutagenesis was performed by the QuickChange method (Agilent) for the Cphy1178 active site mutants. All constructs were sequence confirmed by Sanger sequencing at the Edinburgh Genomics facility.

### Protein production

Plasmids for recombinant aldehyde dehydrogenases were transformed into chemically competent *Escherichia coli* BL21(DE3) cells as follows, 1 μl of plasmid (100 ng/ul) was mixed with 50 μl cells and incubated on ice for 30 minutes. Cells were subsequently subjected to heat shock for 45 seconds at 42 °C and returned to ice for 2 minutes. 200 μl LB media was added to cells before incubation at 37 °C for 1 hour. Cells were then plated onto LB agar supplemented with 10 μg/ml kanamycin and incubated at 37 °C overnight.

Single colonies of transformed cells were transferred to 500 μl LB medium supplemented with 10 ug/ml kanamycin and incubated with shaking to OD_600_ at 37 °C. Cells were induced with 1 mM IPTG and grown overnight at 18 °C. Cells were harvested by centrifugation at 4,000 × *g* for 30 minutes, pelleted cells were washed in PBS and pelleted by centrifugation at 4,000 × *g* for 15 minutes.

### Protein purification

#### Purification of Hexa-histidine tagged proteins

Cells expressing hexa-histidine tagged proteins were resuspended in 10 ml/g (wet weight) buffer HisA (50 mM Tris.HCl, pH 8.0, 500 mM NaCl, 50 mM imidazole). Cells were lysed by sonication on ice, with pulses of 10 μm amplitude for 10 seconds with 10 seconds between pulses for 5 minutes. The resulting cell lysate was clarified by centrifugation at 35,000 × *g* for 30 minutes and the resulting supernatant was filtered with a 0.45 μm syringe filter (Sartorius). Clarified lysate was loaded onto a 5 ml HisTrap column (GE Healthcare) equilibrated with buffer HisA on an Åkta FPLC system (GE Healthcare). Unbound protein was washed from the column with HisA until the A_280_ returned to the baseline value. His_6_-tagged protein was eluted from the column with a step gradient of 5 column volumes of 50% HisA/ 50% HisB (50 mM Tris.HCl, pH 8.0, 500 mM NaCl, 500 mM Imadazole), followed by 5 column volumes of 100% HisB. Protein fractions were analysed by 15% SDS-PAGE to identify the fractions containing the protein of interest. These fractions were loaded onto an S200 16/60 size-exclusion gel-filtration column (GE Healthcare) equilibrated with buffer GF (50 mM Tris.HCl, pH 8.0, 150 mM NaCl) on an Åkta FPLC system. Fractions corresponding to recorded A_280_ peaks were analysed by SDS-PAGE and those containing the protein of interest were pooled for subsequent experiments. A protein purification summary is shown in supplementary table 1.

#### Purification of untagged proteins

Cells expressing untagged proteins were resuspended in 10 ml/g (wet weight) buffer Q-A (50 mM Tris.HCl, pH 8.0) and lysed and clarified as detailed above for His_6_-tagged proteins. Clarified lysate was loaded on a 12 ml Q-sepharose column (GE Healthcare) equilibrated with buffer Q-A. Unbound protein was washed from the column with buffer Q-A until the A_280_ returned to the baseline value. Bound proteins were eluted from the column with a 0–80% linear gradient of 25 column volumes of buffer Q-B (50 mM Tris.HCl, pH 8.0, 1 M NaCl), followed by 5 column volumes of 100% Q-B. Protein fractions were analysed by 15% SDS-PAGE to identify those containing the protein of interest. Fractions containing the protein of interest were pooled and subjected to size-exclusion gel-filtration as described above for His_6_-tagged protein. Fractions containing protein of interest were pooled for subsequent experiments. A protein purification summary is shown in supplementary table 1.

#### Aldehyde dehydrogenase assay

Kinetic assays were performed using a multimode plate reader (Molecular Devices, M5), in flat bottomed 96 well plates (Corning) at 21 °C. Each 300 μl reaction contained 100 mM Tris.HCl, pH 8.0; 0.66 mM NAD^+^; 100 mM KCl; 10 mM 2-mercaptoethanol; 100 nM protein; and various concentrations of aldehyde substrates, using both 100 mM and 10 mM stock solutions to increase assay accuracy. The enzyme activity was monitored by measuring the formation of NADH at 340 nm using an absorption coefficient of 6.22 mM^−1^ cm^−1^. Enzyme activity is expressed as mM NADH produced per second. Steady state kinetic data, obtained with five technical repeats, were analysed by non-linear regression to the Michaelis-Menten equation using GraphPad Prism. Datasets for these assays are available at http://doi.org/10.6084/m9.figshare.2067375.

#### Detection of Propionyl-CoA product formation by LC-MS

To confirm formation of propionyl-CoA, assay mixtures (50 nM Cphy1178_(20–462)_ (reduced with 4 times excess 2-mercaptoethanol and subsequently dialysed into 4000 times excess buffer GF), 75 μM NAD^+^, 100 μM CoA, 10 mM propionaldehyde, 100 mM Tris.HCL pH8.0, 100 mM KCl) were prepared and incubated at room temperature for 2 hours before analysis by LC-MS. Analysis was performed on an ESI Synapt-G2 Q-ToF mass spectrometer equipped with an Acquity UPLC system. For chromatography, samples were separated on a reverse phase Kinetixs C18 50 × 2.1 mm column (Phenomenex), using a gradient of 5 to 95% methanol (0.1% formic acid) over 12 minutes and a flow rate of 250 ul/min. For detection of propionyl-CoA, single ion monitoring was employed with the mass resolving quadrupole set to 824 m/z. The resulting mass spectra were calibrated by applying a single point calibration using Leucine Enkephalin as a reference.

#### Protein mass spectrometry

All mass spectrometry (MS) experiments were performed on a Synapt G2 ion-mobility equipped Q-ToF instrument (Waters). LC-MS experiments were performed using an Acquity UPLC equipped with a reverse phase C4 Aeris Widepore 50 × 2.1 mm HPLC column (Phenomenex) and a gradient of 5_–_95% MeOH (0.1% Formic Acid) over 10 minutes was employed. Samples were typically analysed at 5 μM, and data analysis was performed using MassLynx v4.1 and MaxEnt deconvolution.

For native MS and ion-mobility mass spectrometry (IM-MS) experiments, nano-ESI ionisation was performed using a Nanomate robot operating in infusion mode (Advion Biosciences). Immediately prior to analysis, protein samples were buffer-exchanged into 100 mM ammonium acetate (pH 7.0) using micro BioSpin 6 columns (Biorad). Instrument parameters were carefully tuned to preserve protein complexes and a backing pressure of 4 mbar was used. For IM-MS helium was used as the drift gas. Typically, the IMS wave velocity was set to 300 m/s; wave height to 15 V; and the IMS pressure was 1.8 mbar. For collision cross section determination, IM-MS data was calibrated using denatured equine myoglobin and data was processed using Driftscope v2.5 and MassLynx v4.1 (Waters Corp., UK). For gas-phase disassembly experiments, collision induced dissociation (CID) was employed by applying a Trap collision cell voltage of 75 to 100 V. For solution phase disassembly experiments, The protein tetramer was disrupted in solution by addition of 40% methanol (v/v) prior to MS analysis. Theoretical collision cross sections (CCS) were calculated from PDB files using IMPACT software v. 0.9.1 39.

#### Protein crystallisation and data collection

Purified Cphy1178_(20–462)_ and the C269A and H378A mutants were concentrated to between 8-12 mg/ml in 30 mM NaCl, 50 mM Tris.HCl pH8.0 and crystallised by sitting drop vapour diffusion in drops of 2 μl protein plus 2 μl crystallisation solution, over 1 ml of the latter. Crystals were obtained in 0.1 M sodium acetate pH 4.7, 1.8 M ammonium sulphate and grew as bi-pyramids of 50–100 μm in size. Crystals were harvested from the well using a LithLoop (Molecular Dimensions), transferred briefly to a saturated ammonium sulphate solution and then paratone oil (Molecular Dimensions), excess oil was wicked off and the crystals were subsequently flash cooled in liquid nitrogen. Structures of Cphy1178_(20–462)_ with NADH and CoA ligands were obtained by soaking harvested crystals in a solution of 0.1 M sodium acetate pH 4.7, 1.8 M ammonium sulphate, supplemented with 10 mM ligand and cryo-protected as described above. All crystallographic datasets were collected on beamlines I02 and I04 at Diamond Light Source (Didcot, UK) at 100 K and using ADSC CCD, or Pilatus 6 M detectors. Diffraction data were integrated and scaled using XDS^40^ and merged with Aimless^41^. Data collection and refinement statistics are shown in Table 3.

#### Structure solution and analysis

All structures were solved by molecular replacement using Phaser^42^, using PDBID:3K9D as the molecular replacement model. Refinement of the coordinates, TLS parameters and atomic temperature factors was carried out using Phenix.refine in the Phenix suite^43^. Rounds of iterative model building were performed using Coot^44^. The secondary structure and stereochemistry of the models were analysed using MolProbity^45^. Cofactors present in CoA and NAD^+^ soaked structures were subjected to occupancy refinement for the whole molecule and individual B-factors were refined to accommodate positional uncertainty, rather than using the alternative strategy of setting the occupancy of disordered regions to zero. The oligomeric states of the protein and corresponding buried surface areas were calculated using the PISA server^29^. Structural superimpositions were calculated using Coot. Crystallographic figures were generated with PyMOL (www.pymol.org).

#### Bioinformatics analysis of BMC localisation sequences

Sequences of encapsulated aldehyde dehydrogenase enzymes homologous to Cphy1178 were aligned using Clustalω^46^ and an alignment figure produced using ESPript^47^. Sequence regions falling outside the conserved domain of non-encapsulated aldehyde dehydrogenase enzymes were identified as putative BMC localisation sequences and omitted from the constructs used to produce the aldehyde dehydrogenase proteins for crystallization and activity assays.

## Additional Information

**How to cite this article**: Tuck, L. R. *et al*. Insight into Coenzyme A cofactor binding and the mechanism of acyl-transfer in an acylating aldehyde dehydrogenase from *Clostridium phytofermentans*. *Sci. Rep*. **6**, 22108; doi: 10.1038/srep22108 (2016).

## Supplementary Material

Supplementary Information

## Figures and Tables

**Table 1 t1:** The catalytic activity of Cphy1178 against aldehyde substrates.

Substrate	*k*_cat_ (s^−1^)	*K*_M_(mM)	*k*_cat_/*K*_M_(s^−1^ mM^−1^)	*K*_*i*_(mM)
Acetaldehyde	1.62 ± 0.02	5.16 ± 0.10	0.31	n/a
Propionaldehyde	3.45 ± 0.03	0.82 ± 0.02	4.21	17.31 ± 0.46
Butyraldehyde	3.44 ± 0.7	1.74 ± 0.07	1.98	144.3 ± 30.2
Pentanaldehyde	5.44 ± 0.10	2.2 ± 0.7	2.47	12.61 ± 0.46
Hexanaldehyde	5.61 ± 0.12	2.90 ± 0.10	1.93	23.52 ± 1.28

(Values for *k*_cat_, *K*_*M*_ and *K*_i_ shown calculated standard errors) (See Supplementary Fig. 3 for kinetics curves).

**Table 2 t2:**
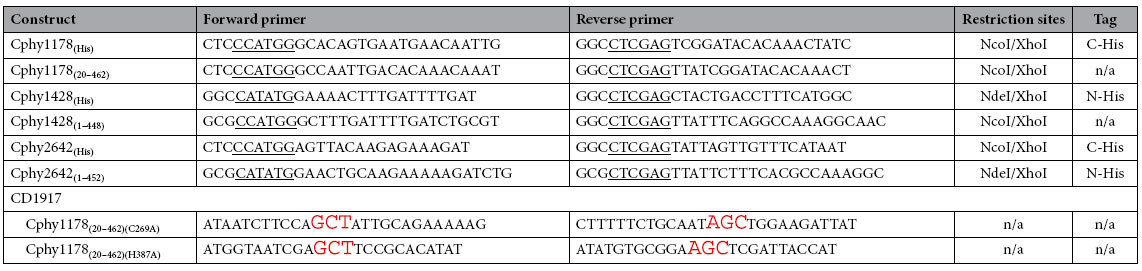
Primers for constructs used in this study.

Primer sequences for constructs used in this work. All primers are listed 5′ to 3′, from left to right. Introduced restriction sites are shown underlined; regions complimentary to genomic DNA shown in bold; sequence mismatches in mutagenic primers are shown in red. All primers are listed 5′ to 3′, from left to right.

**Table 3:** Crystal parameters and data collection statistics for *C. phytofermentans* Cphy1178.

	Cphy1178_(20–462)_		
	NAD^+^	C269A-CoA	H387A
Data Collection			
Wavelength (Å)	0.9795	0.9795	0.9700
Resolution range (Å)	32.64–1.64 (1.67–1.64)	49.89–1.77 (1.8–1.77)	40.53–2.083 (2.158–2.083)
Space group	I 4_1_ 2 2	I 4_1_ 2 2	I 4_1_ 2 2
Unit cell (Å)	138.50, 138.50, 84.60	138.30, 138.30, 84.45	138.45, 138.45, 84.75
Total reflections	299,010 (14,770)	518,921 (25,397)	24,2393 (23,123)
Unique reflections	50,131 (24,674)	40,079 (2229)	24,570 (2,389)
Multiplicity	6.0 (6.0)	12.9 (11.4)	10.0 (9.7)
Completeness (%)	99.7 (99.9)	99.9 (98.5)	98.85 (99.25)
Mean I/sigma(I)	17.8 (2.3)	22.4 (2.3)	18.94 (1.63)
Wilson B-factor (Å^2^)	16.52	24.95	40.85
R_merge_	0.062 (0.697)	0.073 (1.050)	0.081 (1.227)
R_meas_	0.074	0.079 (1.152)	0.08496
CC1/2	0.999 (0.744)	0.999 (0.738)	1 (0.649)
Diffraction images (DOI)	10.7488/ds/1307	10.7488/ds/1318	10.7488/ds/1319
Model Building and Refinement			
R_work_	0.1463 (0.2110)	0.1465 (0.2122)	0.1823 (0.2651)
R_free_	0.1738 (0.2376)	0.1830 (0.2682)	0.2575 (0.3236)
Number of non-hydrogen atoms	3795	3597	3376
macromolecules	3293	3225	3245
ligands	69	79	5
water	433	293	126
Protein residues	435	431	435
RMS(bonds) (Å)	0.011	0.010	0.015
RMS(angles) (°)	1.37	1.26	1.44
Ramachandran			
favored (%)	99	99	98
outliers (%)	0	0	0
Clashscore	2.66	0.45	4.42
Average B-factor (Å^2^)	24.90	33.90	56.80
macromolecules	23.90	32.50	57.10
ligands	28.30	79.8	80.8
solvent	32.10	37	46.50
PDBID	4C3S	5DBV	5DRU

Statistics for the highest-resolution shell are shown in parentheses.
