# Supplementary material for: Insight into Coenzyme A cofactor binding and the mechanism of acyl-transfer in an acylating aldehyde dehydrogenase from Clostridium phytofermentans
Source: Sci Rep. 2016 Feb 22;6:22108. doi: 10.1038/srep22108 (PMC4762007; doi:10.1038/srep22108)
Supplement: Supplementary Information [file srep22108-s1.doc]

**Supplementary Information**

**Insight into Coenzyme A cofactor binding and the mechanism of acyl-transfer in an acylating aldehyde dehydrogenase from *Clostridium phytofermentans***

Laura R. Tuck^1^, Kirsten Altenbach^1†^, Thiau Fu Ang^1†^, Adam D. Crawshaw^2^, Dominic J. Campopiano^3^, David J. Clarke^3^, Jon Marles-Wright^1*^

**
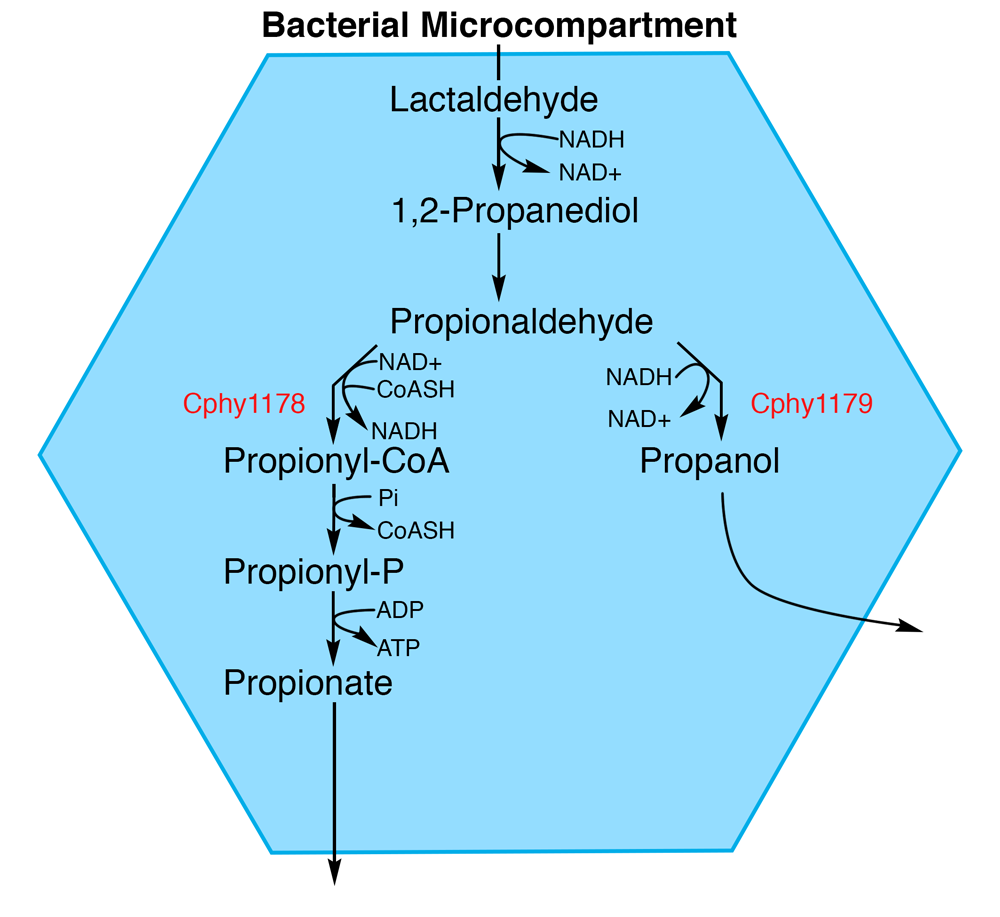
**

**Supplementary Figure 1. Scheme for Clostridium phytofermentans fucose/rhamnose utilisation pathway.** Pathway intermediates and cofactors shown with proposed aldehyde dehydrogenase (Cphy1178) and alcohol dehydrogenase (Cphy1179) shown in red. Scheme based on Petit et al^1^.

**
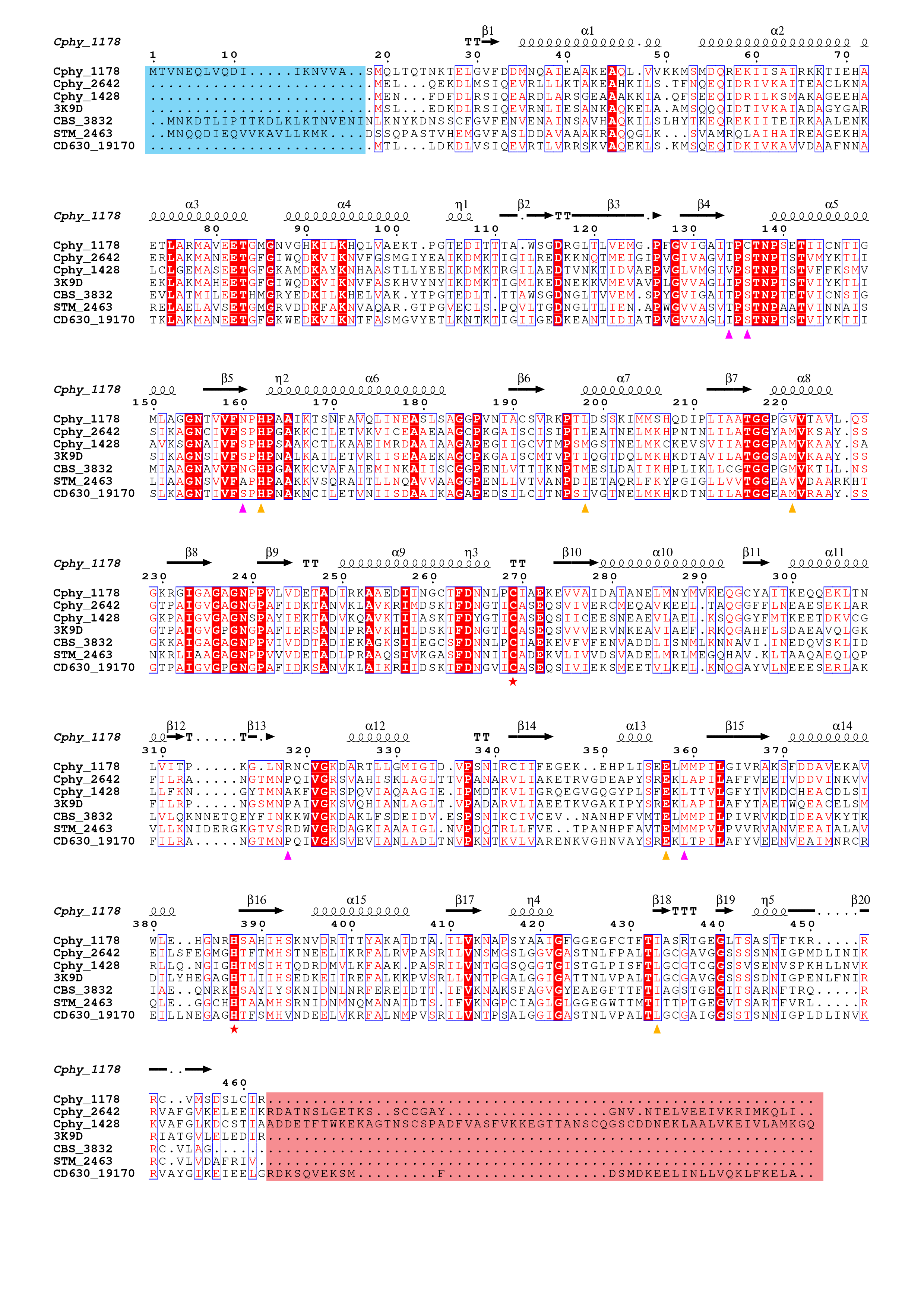
**

**Supplementary Figure 2. Multiple sequence alignment of aldehyde dehydrogenase enzymes used in this study and close homologues.** Sequence alignment generated using ClustalΩ^2^ and displayed using ESPript^3^. Putative N- and C-terminal BMC localisation sequences are shown as blue and red boxes respectively. Secondary structure elements of Cphy_1178 are shown above the alignment and cofactor binding residues labelled with orange triangles for NAD and pink triangles for CoA, the catalytic cysteine and histidine residues are labelled with red stars.

**Supplementary Figure 3. Activity of Cphy1178 against various aldehyde substrates.** Cphy1178 was incubated with varying concentrations of aldehyde substrates, with the following additional components in the reaction mixture: 100 mM Tris.HCl (pH 8.0); 0.66 mM NAD^+^, 100 mM KCl, 10 mM 2-mercaptoethanol. Data were analysed using Graphpad Prism and kinetic parameters were calculated by non-linear regression to the Michaelis-Menten equation with substrate inhibition for C3 and larger aldehydes. (Velocity is expressed as mM NADH s^-1^ mM^-1^ADH).

**
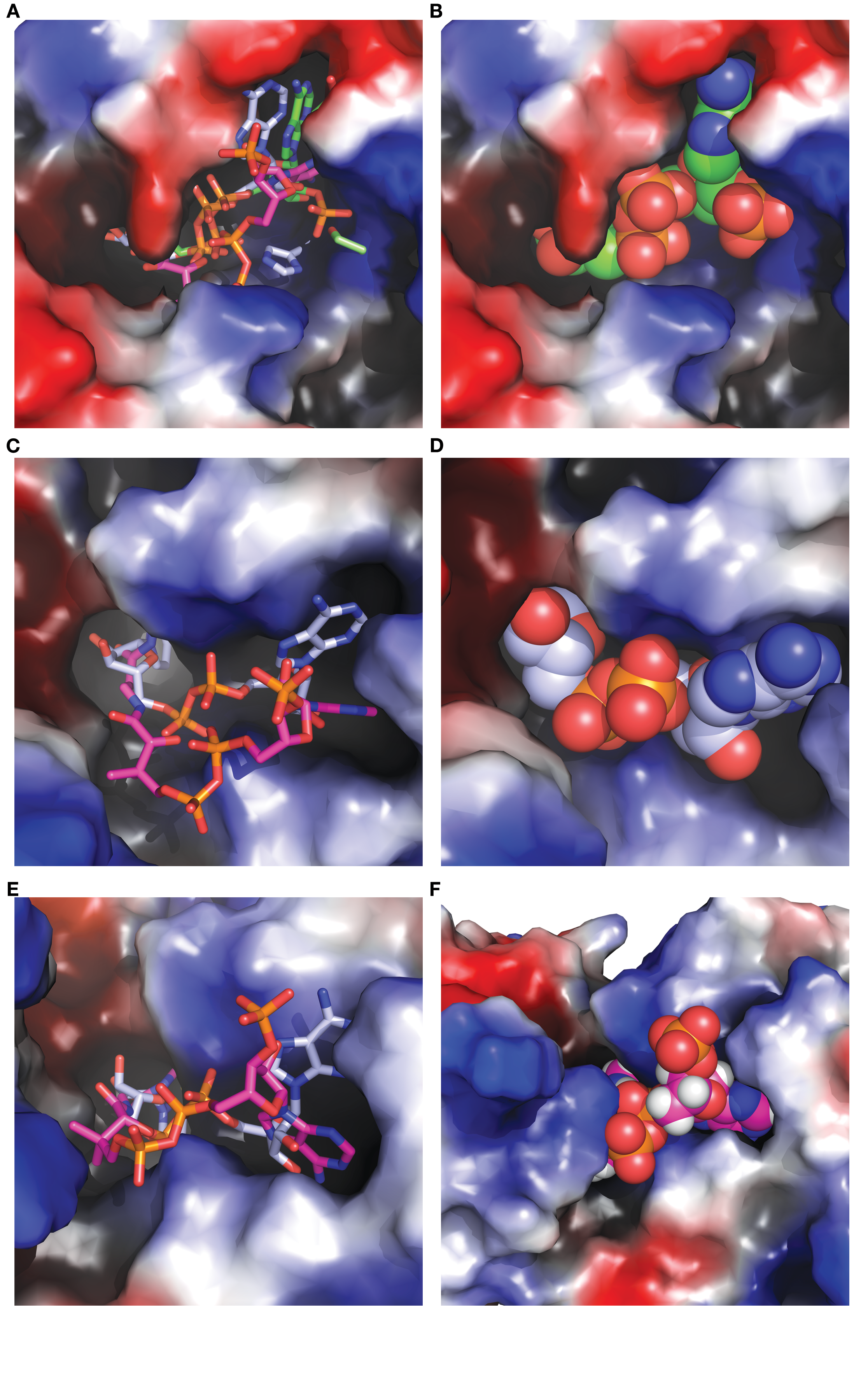
Supplementary Figure 4.** Surface views of cofactor binding to Cphy1178_20-462_. (A) Comparison of cofactor positions in Cphy1178_20-462_ with NADP^+^ position in PDBID:4NMJ. Electrostatic surface shown for 4NMJ and carbon atoms of NADP^+^ shown in green. His162 and P163 of Cphy117820-462 shown as stick representations. (B) Spacefilling view of NADP^+^ bound to 4NMJ. (C) Top view of adenine binding pockets in Cphy1178_20-462_. NAD^+^ shown with blue carbon atoms, CoA shown with magenta carbons. (D) Spacefilling representation of NAD^+^. (E) Oblique view of adenine binding pockets depicted as in (C). (F) Spacefilling representation of CoA.


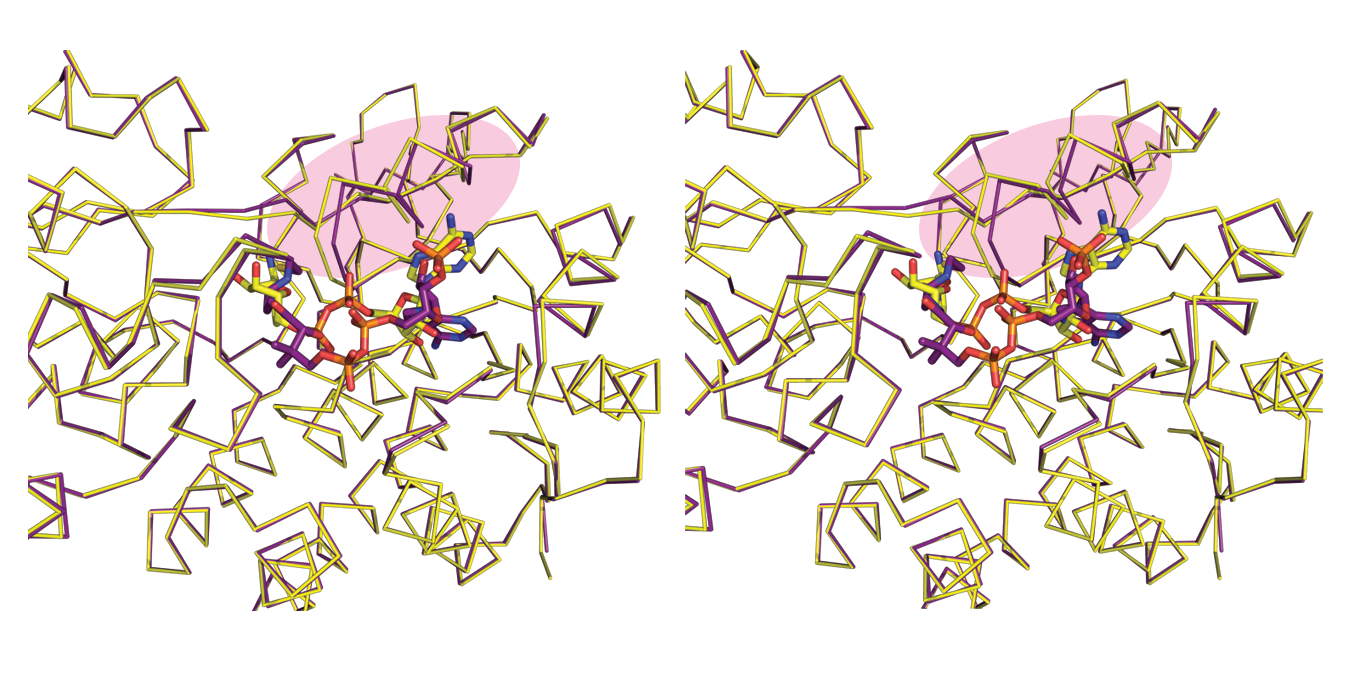
**Supplementary Figure 5.** Wall-eyed stereo view of cofactor binding within the nucleotide-binding domain of Cphy1178_20-462_. Ribbon representation of the proteins and stick representations of the NAD^+^ and CoA cofactors are displayed to show the minor differenced between the two cofactor bound structures of Cphy1178_20-462_ the helix highlighted in pink, between residues 215 and 225 is shifted slightly in the CoA bound structure (purple) when compared to the NAD^+^ bound structure.

**Supplementary Table 1. Protein purification summary for Cphy_1178_His6_**

| **Step** | **Total protein**  **(mg)^b^** | **Total activity**  **(mM NADH min^-1^)^c^** | **Specific activity**  **(mM NADH min^-1^ mg^-1^)** | **Yield**  **(%)** | **Fold purification** |
| --- | --- | --- | --- | --- | --- |
| Crude lysate^a^ | 82.3 | 62.7 | 0.76 | 100 | 1 |
| Crude extract | 35.6 | 84.6 | 2.38 | 135 | 3.13 |
| HiTrap eluate (pooled peak) | 5.75 | 29.142 | 5.07 | 46 | 6.67 |
| S200 gel filtration (pooled peak) | 1.48 | 6.78 | 4.58 | 10 | 6.02 |

^a^ From 1.7g wet weight *E. coli* cell pellet from 0.5 litres bacterial culture.

^b^ Protein concentration determined by BCA method using BSA as a standard protein.

^c^ Enzyme activity measured as described in the methods section.

**Supplementary Table 2. Protein purification summary for CPhy_1178_20-426_**

| **Step** | **Total protein**  **(mg)^b^** | **Total activity**  **(mM NADH min^-1^)^c^** | **Specific activity**  **(mM NADH min^-1^ mg^-1^)** | **Yield**  **(%)** | **Fold purification**  **(%)** |
| --- | --- | --- | --- | --- | --- |
| Crude lysate^a^ | 71 | 74.52 | 1.05 | 100 | 1 |
| Crude extract | 33.3 | 98.22 | 2.95 | 138 | 2.8 |
| Q-sepharose eluate (pooled peak) | 5.89 | 12.6 | 2.13 | 16.9 | 2.02 |
| S200 gel filtration (pooled peak) | 0.53 | 3.582 | 6.76 | 4 | 6.38 |

^a^ From 1.2g wet weight *E. coli* cell pellet from 0.5 litres bacterial culture.

^b^ Protein concentration determined by BCA method using BSA as a standard protein.

^c^ Enzyme activity measured as described in the methods section.

**Supplementary References**

1. Petit, E. *et al.* Involvement of a Bacterial Microcompartment in the Metabolism of Fucose and Rhamnose by Clostridium phytofermentans. *PLoS One* **8,** e54337 (2013).

2. Sievers, F. & Higgins, D. G. Clustal Omega, accurate alignment of very large numbers of sequences. *Methods Mol. Biol.* **1079,** 105–16 (2014).

3. Gouet, P., Robert, X. & Courcelle, E. ESPript/ENDscript: Extracting and rendering sequence and 3D information from atomic structures of proteins. *Nucleic Acids Res.* **31,** 3320–3 (2003).
